# GPCRDB: an information system for G protein-coupled receptors

**DOI:** 10.1093/nar/gkt1255

**Published:** 2013-12-03

**Authors:** Vignir Isberg, Bas Vroling, Rob van der Kant, Kang Li, Gert Vriend, David Gloriam

**Affiliations:** ^1^Department of Drug Design and Pharmacology, University of Copenhagen, Universitetsparken 2, DK-2100 Copenhagen, Denmark, ^2^Bio-Prodict B.V., Castellastraat 116, 6512 EZ, Nijmegen, The Netherlands and ^3^CMBI, NCMLS, Radboudumc Nijmegen Medical Centre, Geert Grooteplein Zuid 26-28, 6525 GA, Nijmegen, The Netherlands

## Abstract

For the past 20 years, the GPCRDB (G protein-coupled receptors database; http://www.gpcr.org/7tm/) has been a ‘one-stop shop’ for G protein-coupled receptor (GPCR)-related data. The GPCRDB contains experimental data on sequences, ligand-binding constants, mutations and oligomers, as well as many different types of computationally derived data, such as multiple sequence alignments and homology models. The GPCRDB also provides visualization and analysis tools, plus a number of query systems. In the latest GPCRDB release, all multiple sequence alignments, and >65 000 homology models, have been significantly improved, thanks to a recent flurry of GPCR X-ray structure data. Tools were introduced to browse X-ray structures, compare binding sites, profile similar receptors and generate amino acid conservation statistics. Snake plots and helix box diagrams can now be custom coloured (e.g. by chemical properties or mutation data) and saved as figures. A series of sequence alignment visualization tools has been added, and sequence alignments can now be created for subsets of sequences and sequence positions, and alignment statistics can be produced for any of these subsets.

## INTRODUCTION

G protein-coupled receptors (GPCRs) constitute a large family of cell surface receptors. They regulate a wide range of cellular processes, including those associated with taste, smell and vision, and they control myriad intracellular systems, ranging from neurotransmission to hormone signalling. GPCRs are major targets for the pharmaceutical industry, as reflected by the fact that more than a quarter of all FDA-approved drugs act on a GPCR (1). At present, only ∼30 of the ∼350 genes that code for non-olfactory receptors in the human species (2) are truly validated therapeutic targets (3), indicating this family’s immense potential for future drug development. An increasing number of drugs have been found to display polypharmacology, i.e. activity through multiple receptor targets (4). However, endogenous ligands for ∼135 of the so-called orphan receptors have so far eluded researchers.

Early releases of the GPCRDB (5–8) focused on the compilation and homogeneous presentation of many types of heterogeneous data, with the aim of providing the four main facilities needed in an information system: browsing, querying, retrieval and inference. The first three of these facilities have been available ever since the start of the project, but received a major boost when the GPCRDB was coupled to an intelligent PDF reader (9) that puts all relevant aspects of the GPCRDB non-intrusively in a side-bar in the PDF reader window. Inference really only started when a number of interactive tools were added (10), enabling bioinformaticians to interact with multiple sequence alignments, together with derived data such as entropy and variability scores, in an integrated environment. For example, these tools were successfully applied in the 2010 GPCR-Dock competition (11). In the past, computing facilities were aimed at expert GPCR bioinformaticians. In contrast, the new interactive tools are readily accessible to non-expert users and allow faster execution of visualization and analysis tasks.

## STRUCTURAL DATA AND TOOLS

### Crystal structure browser

In recent years, X-ray crystallography of GPCRs has revealed the sites and mechanisms for binding ligands, lipids, and G proteins, as well as the conformations of activated states. The GPCRDB structure browser includes manually annotated key data for all ligand–receptor complexes and at least one (the highest resolution) representative file for receptors solved only as apo structures. The structure browser can select crystal structures based on a series of filters for receptors, ligands, activation states, G protein presence, PDB and Pubmed identifiers, resolution, structure completeness and so forth. Sequence similarities can be retrieved by specifying a receptor reference, facilitating template selection for homology modelling.

### Structure-based sequence alignments and homology models

**Figure 1.**
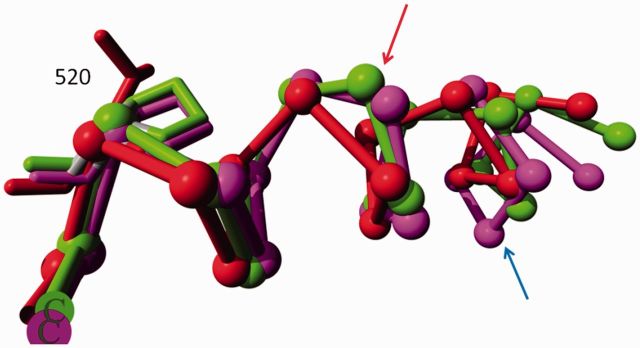
The area around the bulge in helix V. The S1P lipid receptor [red, PDBid = 3v2y (12)] does not have α-bulges in helix V and is provided as a reference. Rhodopsin [green, PDBid = 1f88 (13)] and the adenosine-2A receptor [purple, PDBid = 3eml (14)] have an α-bulge (red arrow) between positions 516^5.46^ and 517^5.47^. The adenosine-2A receptor has an extra bulge (blue arrow) between positions 511^5.41^ and 512^5.42^. Rhodopsin and the adenosine-2A receptor have a proline at position 520^5.50^. The S1P lipid receptor, which does not have bulges in helix V, does not have a proline at position 520^5.50^. Time will tell whether this correlation is accidental or causal. Residues are numbered using the GPCRDB scheme with the B&W numbers given as superscripts.

**Figure 2.**
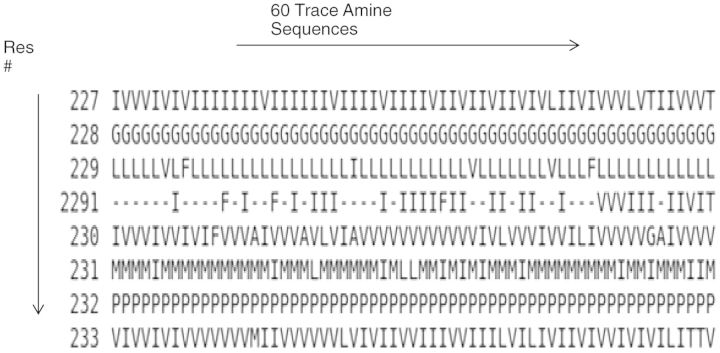
Alignment of the middle part of TM helix II in 60 trace amine receptors. The residues 227^2.57^–233^2.63^ (GPCRDB numbers with B&W numbers as superscripts) are shown running vertically using the GPCRDB numbering scheme.

GPCR transmembrane (TM) helices are known to contain many irregularities. However, it was only recently realized, with the availability of new crystallographic data, that GPCRs contain many more *α*-bulges than those in helix II and helix V. The *α*-bulges in GPCRs are relatively non-conserved. As an example of this non-conservation, Figure 1 shows the *α*-bulges in helix V of rhodopsin and the adenosine 2A receptor, and Figure 2 shows the alignment of the middle part of helix II in 60 trace amine receptors. The receptors are on average 70% sequence identical, but the bulge is only present in around half of all family members.

The abundance of *α*-bulges required a novel residue numbering approach that involved rewriting the DSSP secondary structure analysis software (15) and the GPCR-specific alignment software. This, in turn, meant that >65 000 homology models had to be constructed again. All GPCR sequences are now modelled twice-once using as template the most sequence-similar inactive form, and once using the most similar active form. Modelling was done with YASARA (16), aligning the model with the template contained in the GPCRDB and using default values for all other parameters and options. All GPCR alignment profiles were manually updated to reflect our latest knowledge about the *α*-bulges, and all 1272 alignments were regenerated. It seems likely that this exercise will need to be repeated in the coming years as new GPCR structure data reveal new *α*-bulge patterns.

### Translation of generic and receptor-specific residue numbers

We wanted to take proper care of the *α*-bulges that are widely present in six of the seven GPCR TM helices without excessively changing the commonly used generic residue numbering schemes. The Oliveira numbering (17) and the B&W numbers (18) were maintained as far as possible, whereas bulge residues were given the same number as the residue directly N-terminal in the sequence but with a digit added that reflects the number of the bulge. The Utopia-GPCRDB PDF reader automatically takes these new numbers into account, whereas our new sequence indexing tool provides computational access to all generic and receptor-specific residue numbers for selected receptors.

## RECEPTOR SEQUENCE DIAGRAMS

### Snake-like and helix box diagrams

**Figure 3.**
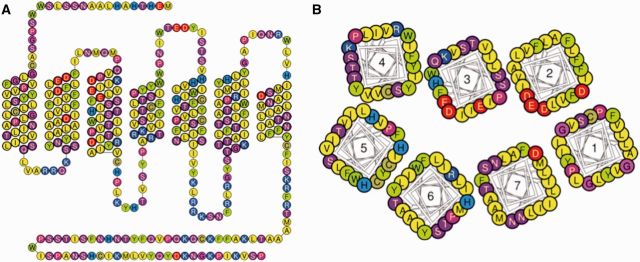
Snake-like (**A**) and helix box (**B**) diagrams depict GPCRs from the side and top, respectively.

Snake-like diagrams now include the full loops and terminal sequences (Figure 3A). New helix box diagrams present the TM helices as seen from ‘above’ (Figure 3A). These diagrams are similar to previously used helical wheel plots but orient the amino acids in better agreement with the 3D structures. Hovering the computer mouse pointer over the TM amino acids displays their residue numbers. Amino acids can be coloured to illustrate their physicochemical properties, or the presence of mutation data or the mutation effects. The diagrams can be downloaded as picture files or in scalable vector graphics format to allow further editing.

### Residue conservation and property statistics

In functional or evolutionary studies of specific amino acids or their properties, it is typically relevant to find out which additional receptors share the conserved amino acids or properties (e.g. specific charge or hydrophobicity). Therefore, sequence alignments are augmented with a series of statistics. For each position, the following data are listed: the consensus sequence, the percentage of each of the 20 amino acids present, and percentages for relevant properties such as aromaticity, acidity or hydrogen bonding capability.

## (BINDING) SITE-SPECIFIC RECEPTOR SIMILARITIES

### Alignment sub-site/domain selections

The alignment and similarity tools integrated into GPCRDB offer users the ability to select arbitrary combinations of helices, residue positions or predefined sets of residues, for example, the amino acids in the TM binding cavity (19). By focusing on a given functional site rather than the full sequence, the receptor similarities will better reflect the structural features involved in, for example, receptor dimerization, ligand binding or G protein binding.

### Similarity search with a reference receptor (one-to-all similarities)

Similarity searches are conducted by specifying a target of interest, a set of receptors and the residue positions of interest. Results are presented as a sequence alignment, in which the target is followed by a list of hits in order of sequence identity, similarity or alignment score. The data can be downloaded as either an alignment file or a spreadsheet.

### Trees (all-to-all similarities)

Neighbour-joining trees (20) can be generated based on any sub-site/domain and set of receptors. Trees can be calculated with up to 100 bootstraps, displayed in circular and ladder representations, and downloaded in Newick format for use with stand-alone tree software.

### Sequence motif search (conserved and non-conserved separation)

The sequence motif search tool generates more precise and discriminative results, by allowing residues to be matched for relevant amino acid properties (21), e.g. their hydrophobicity, hydrogen bond donor capability or size. Relevant applications for this tool include rationalization of observed polypharmacology, receptor panel selection for off target screening and ligand inference from old to new targets.

## CONCLUSIONS

The 20th yearly release of the GPCRDB includes a large number of novel discoveries. The solved structures (see http://gpcr.scripps.edu/) reveal the presence of many *α*-bulges that are not conserved among or even within GPCR subfamilies. We have updated all the alignments and homology models, together with the residue numbering schemes, to ensure agreement between the contents of the GPCRDB and new insights obtained by studying all the available structure data. Additionally, the new GPCRDB release includes a powerful yet user-friendly computational toolbox that provides users with crystal structure browser, receptor visualization and alignment analysis tools, plus options to study receptor similarity both quantitatively and graphically.

## FUNDING

Lundbeck Foundation [R54-A5441 to V.I.]; the Carlsberg Foundation [R77-A6854 to D.G.] and the European Union NewProt project [289350 to G.V.]. Funding for open access charge: The publication will be paid by the CMBI running costs.

*Conflict of interest statement*. None declared.
